# Comparative Analysis of Clavien–Dindo Grade and Risk Factors of Complications after Dual-Port Laparoscopic Distal Gastrectomy and Hand-Assisted Laparoscopic Gastrectomy

**DOI:** 10.1155/2021/4747843

**Published:** 2021-07-09

**Authors:** Haihao Jin, Jianshan Geng

**Affiliations:** Department of Gastroenterology, Laiyang People's Hospital, Laiyang 265200, Shandong, China

## Abstract

**Objective:**

To compare the Clavien–Dindo grade and risk factors of complications after dual-port laparoscopic distal gastrectomy (DPLDG) and hand-assisted laparoscopic gastrectomy (HALG).

**Methods:**

The clinical data of 775 patients who underwent DPLDG or HALG in our hospital from May 2016 to May 2019 were retrospectively reviewed, and the patients were divided into the DPLDG group (*n* = 386) and HALG group (*n* = 389) according to the surgical method to explore the risk factors of postoperative complications by grading their postoperative complications according to the Clavien–Dindo classification system and single-factor and multivariate analysis of the association between variables in clinical data and complications.

**Results:**

Compared with the HALG group, the DPLDG group had significantly shorter surgical time, less intraoperative blood loss, and better postoperative exhaust time (*p* < 0.05), with no significant difference in other clinical indicators between the two groups (*p* > 0.05); the postoperative complication incidence rate of DPLDG group was significantly lower than that of the HALG group; it was shown in the single-factor analysis that the age, tumor length, intraoperative blood loss, pathological stages, and surgical method were related to the postoperative complications, and the results of multivariate analysis indicated that DPLDG was the protective factor for reducing postoperative complications, while age no less than 60 years old and intraoperative blood loss no less than 180 ml were the independent risk factors leading to complications; after surgery, the PNI level values at T1, T2, and T3 of DPLDG group were significantly higher than those of the HALG group (*p* < 0.05); and at 1 month after surgery, both groups obtained significantly higher GLQI scores than before, and the GLQI score of the DPLDG group was significantly higher in the between-group comparison (*p* < 0.05).

**Conclusion:**

The DPLDG has lower postoperative complication incidence rate than the HALG, but age no less than 60 years old and intraoperative blood loss not less than 180 ml are the independent risk factors for postoperative complications, so advanced prevention measures shall be taken to lower the incidence of complications.

## 1. Introduction

Gastric cancer is a kind of gastroenterological cancer originating from the gastric epithelial cell, which, according to related statistics [1, 2], ranks the fifth in the incidence and top three in the mortality among the new cancers worldwide. With the gradual development of clinical research, people have a deeper understanding of gastric cancer, and laparoscopy with the characteristics of less trauma and precise operative manipulation has been widely used in the treatment of gastric cancer, which can effectively remove lymph nodes and improve the survival outcome of patients [3]. In recent years, the totally laparoscopic distal gastrectomy (TLDG) has become more widely applied in the clinic, and studies have confirmed that TLDG has comparable long-term outcomes compared with open surgery. As an important branch of TLDG, hand-assisted laparoscopic gastrectomy (HALG) combines the advantages of TLDG with those of open surgery, thereby exerting the best therapeutic effect against gastric cancer [4–6]. In traditional TLDG, 5 ports plus auxiliary small incision reconstruction are usually needed, but with the continuous innovation and development of minimal invasive technique, laparoscopic gastrectomy with less ports is applied. Theoretically speaking, with fewer ports, the operation will be less difficult and the patients are less traumatized. However, as D2 lymphadenectomy is difficult in surgery and the operation with single port is more complicated than that with 5 ports, the single-port laparoscopic radical gastrectomy is limited in application [7, 8]. On the basis of the single-port laparoscopy, the two-port method is conducted by adding an auxiliary operation port in the patient's right upper abdomen for laparoscopic drainage, which reduces trauma and lowers the difficulty of operation while not increasing the abdominal incision, which has been demonstrated in the clinical treatment of gastric cancer [9]. Previous literature is short of studies on Clavien–Dindo grade and risk factors of complications after DPLDG and HALG [10]. Based on this, the clinical data of 775 patients who underwent DPLDG or HALG in our hospital were retrospectively reviewed to analyze the effect of different surgical procedures on the Clavien–Dindo grade and risk factors of postoperative complications.

## 2. Materials and Methods

### 2.1. General Information

The clinical data of 775 patients who underwent distal gastrectomy in our hospital from May 2016 to May 2019 were retrospectively reviewed, and the patients were divided into the DPLDG group (*n* = 386) and HALG group (*n* = 389) according to the surgical method, with no significant difference in the baseline information between the two groups (*p* > 0.05), see Table 1.

### 2.2. Inclusion and Exclusion Criteria

#### 2.2.1. Inclusion Criteria

(1) Patients met the clinical diagnosis criteria of gastric cancer in the *Surgery of Gastric Cancer* [11] and were confirmed to have gastric cancer by histopathological examination in vivo; (2) patients were not given chemoradiotherapy before surgery; (3) patients met the surgical indications; and (4) the study was reviewed and approved by the Hospital Ethics Committee, and patients and their family members signed the informed consent. *Exclusion Criteria*. (1) Patients had other malignant tumors; (2) patients had history of abdominal surgery or accepted the palliative resection of tumor before; (3) patients had serious brain, heart, lung, or kidney diseases; and (4) patients had coagulation disorders or serious basic diseases.

### 2.3. Methods

Both surgeries were performed by the same medical team, and the specific steps of DPLDG were as follows. After performing general anesthesia with tracheal intubation, the self-developed single-port multichannel operation device (made by Taizhou Roosin Medical Co., Ltd.) was used to make a 3-4 cm incision below or around the umbilicus on the left side. After the device entered the patient's abdominal cavity layer by layer, a matched incision protective sleeve was placed to hold the device and establish the pneumoperitoneum, with the pressure maintained at 12–15 mmHg. A 5 mm trocar was placed at the subcostal margin of right midclavicular line as the auxiliary operation hole to hang the liver. The ultrasound knife was used to free along the transverse colon to the splenic flexure to fully expose the pancreatic tail and the lower pole of the spleen, position the left gastroepiploic vessel, ligate the left gastroepiploic vessel at the root, and dissect the No. 4sb lymph node. The mesogastrium was separated from the transverse mesocolon, the right gastroomental vein was positioned at the inferior border of pancreas, the right gastroepiploic artery was positioned, and the root was ligated. The No. 6 lymph node was dissected by freeing along the subpyloric region to the suprapyloric region with the gastroduodenal artery as the clue. And the No. 5 lymph node was dissected by dividing the duodenal ampulla with the linear cutting closure and ligating the root. The No. 8a, No. 9, No. 11p, and No. 7 lymph nodes were dissected by freeing the splenic artery, common hepatic artery, and left gastric artery and vein, respectively, at the upper border of pancreas and retropancreatic space and ligating the left gastric vessel, the No. 12a lymph node was dissected by exposing the left portal vein wall, and the No. 3 and No. 1 lymph nodes were dissected after freeing along the lesser curvature of stomach; the gastric body was disconnected at 4-5 cm proximal to the tumor with the linear cutting closure, and the Roux-en-Y anastomosis or Billroth II anastomosis was performed in the cavity. Finally, a drainage tube was indwelled near the gastrointestinal anastomosis port through the auxiliary operation hole in the right upper abdomen.

The specific steps of performing HALG were as follows. General anesthesia was implemented in flat position; a disinfected drape was laid. The operator was standing on the right side of the patient to make an incision in the middle of the upper abdomen and then incised various layers of the abdominal wall sequentially to the abdomen and placed the lap disc base to explore the patient's abdominal cavity in order. The transverse colon was lifted and partial gastrocolic ligament was freed along the upper board of the transverse colon to expose the right crura of diaphragm. A 12 mm trocar was placed as the main operation hole, and the operator put his left hand into the abdominal cavity to install the lap disc and establish a pneumoperitoneum. A 12 mm trocar was placed at about 2 cm of subcostal margin of left anterior axillary line as the observation hole. The lymph node dissection was performed according to the tumor location and the relevant criteria in the latest *radical gastrectomy* [12]. The pneumoperitoneum was closed, and the digestive tract reconstruction was completed under euthyphoria. The Billroth I or II anastomosis was performed on patients who underwent distal gastrectomy, and the Roux-en-Y anastomosis was performed on patients who underwent oesophagus jejunum after total gastrectomy.

### 2.4. Evaluation Indexes

The surgical time, intraoperative blood loss, incision length, postoperative exhaust time, time to return to full-fluid diet for the first time after surgery, hospital stay after surgery, the off-bed ambulation time after surgery, the number of lymph nodes being dissected, and the time of removing the abdominal drainage tube of patients in both groups were recorded. The pain severity of patients in both groups was assessed by the numerical pain rating (NRS) scale [13] at 6 h, 12 h, 24 h, and 2 d postoperatively. The patients' self-evaluation to their incisions was assessed by the aesthetic scoring, which was composed of three parts, namely, the satisfaction of incision scar (1–7 points, with 1 point indicating unsatisfied and 7 points indicating fully satisfied), incision scar (1–7 points, with 1 point indicating disgusted and 7 points indicating enjoyed), and incision scar classification (1–10 points, with 1 point indicating the worst and 7 points indicating the best). The sum of the three parts was the final score of incision aesthetic, which was the basis for grading patients' satisfaction of incision as low (3–9 points), medium (10–17 points), and high (18–24 points).

The complication severity within 30 days after surgery was evaluated by the Clavien–Dindo classification system [14], and if the patient had more than one complication, the most severe one was recorded. IIIa complications or complications with higher grade were specified as severe.

The serum albumin (Alb, g/L) and peripheral blood lymphocyte count (Lymph, mm^3^) of patients in both groups were tested before surgery and at 3 months, 6 months, and 12 months after surgery, and the prognosis nutrition index (PNI) was calculated by the formula (PNI) = (Alb) × 10 + (Lymph) × 0.005. The quality of life of patients in both groups before surgery and at 1 month after surgery was evaluated by the gastrointestinal quality of life index (GQLI) [15]. The maximum score was 145 points, with higher scores indicating better quality of life.

### 2.5. Statistical Methods

The experimental data were statistically analyzed and processed by the SPSS21.0, the picture drawing of data was completed by GraphPad Prism 7 (GraphPad Software, San Diego, USA), the enumeration data were examined by *X*^2^ test and expressed by [*n* (%)], the measurement data were examined by *t*-test and expressed by $\left(x\bar{}\pm s\right)$, the single-factor analysis and multivariate analysis of risk factors of postoperative complications were examined by *X*^2^ test and logistic regression model respectively, and differences were considered statistically significant at *p* < 0.05.

## 3. Results

### 3.1. Comparison of Patients' Clinical Effect Indexes between the Two Groups

Compared with the HALG group, the DPLDG group had significantly shorter surgical time, less intraoperative blood loss, and higher postoperative exhaust time (*p* < 0.05), with no significant difference in other clinical indexes (*p* > 0.05); see Table 2.

### 3.2. Comparison of Patients' Clavien–Dindo Grades of Postoperative Complications between the Two Groups

The overall incidence rate of postoperative complications of the DPLDG group was significantly lower than that of the HALG group (*p* < 0.05); see Table 3.

### 3.3. Single-Factor and Multivariate Analysis of Risk Factors of Postoperative Complications in Patients of Both Groups

It was showed in the single-factor analysis that the age, tumor length, intraoperative blood loss, pathological stages, and surgical method were related to the postoperative complications, and the results of multivariate analysis indicated that DPLDG was the protective factor for reducing postoperative complications, while age no less than 60 years old and intraoperative blood loss no less than 180 ml were the independent risk factors leading to complications; see Table 4.

### 3.4. Comparison of Patients' PNI Level Values before Surgery and at Different Moments after Surgery between the Two Groups

**Figure 1:**
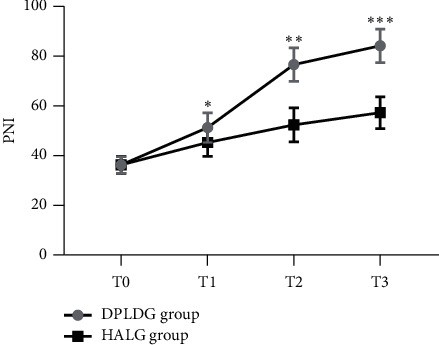
Comparison of patients' PNI level values before surgery and at different moments after surgery between the two groups $\left(x\bar{}\pm s\right)$. *Note*. The horizontal axis indicated T0, T1, T2, and T3, and the vertical axis indicated the PNI level values. The PNI level values at T0, T1, T2, and T3 of patients in the DPLDG group were 36.26 ± 3.37, 51.26 ± 5.97, 76.63 ± 6.74, and 84.17 ± 6.69, respectively. The PNI level values at T0, T1, T2, and T3 of patients in the HALG group were 36.29 ± 3.41, 45.33 ± 5.63, 52.36 ± 6.81, and 57.26 ± 6.37, respectively. ^*∗*^ indicates that the PNI level values at T1 of patients in the two groups were significantly different (*t* = 14.227, *p* < 0.001); ^*∗∗*^ indicates that the PNI level values at T2 of patients in the two groups were significantly different (*t* = 49.861, *p* < 0.001); and ^*∗∗∗*^ indicates that the PNI level values at T3 of patients in the two groups were significantly different (*t* = 57.349, *p* < 0.001).

The PNI level values at T1, T2, and T3 after surgery of patients in the DPLDG group were significantly higher than those in the HALG group (*p* < 0.05); see Figure 1.

### 3.5. Comparison of Patients' GLQI Scores before Surgery and at 1 Month after Surgery between the Two Groups

**Figure 2:**
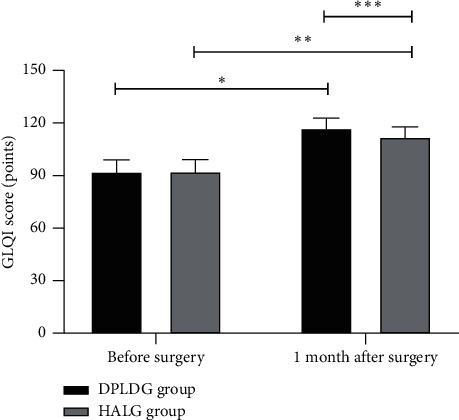
Comparison of patients' GLQI scores before surgery and at 1 month after surgery between the two groups $\left(x\bar{}\pm s\right)$. *Note*. The horizontal axis indicated before surgery and 1 month after surgery, and the vertical axis indicated the GLQI score (points). The GLQI scores before surgery and at 1 month after surgery of patients in the DPLDG group were (92.35 ± 6.74) and (117.36 ± 5.43), respectively, and those of patients in the HALG group were (92.38 ± 6.79) and (112.36 ± 5.49), respectively. ^*∗*^indicates that the GLQI scores before surgery and at 1 month after surgery of patients in the DPLDG group were significantly different (*t* = 56.771, *p* < 0.001); ^*∗∗*^indicates that the GLQI scores before surgery and at 1 month after surgery of patients in the HALG group were significantly different (*t* = 45.130, *p* < 0.001); and ^*∗∗∗*^indicates that the GLQI scores at 1 month after surgery of patients in the two groups were significantly different (*t* = 12.746, *p* < 0.001).

At 1 month after surgery, both groups obtained significantly higher GLQI scores than before, and the GLQI score of the DPLDG group was significantly higher in the between-group comparison (*p* < 0.05); see Figure 2.

## 4. Discussion

Gastric cancer is a malignant tumor disease of the digestive tract. With the higher demand for the surgical efficacy of gastric cancer currently, an ideal surgical treatment regimen that aims to reduce the impact on the patient's body function and prolong survival time is needed [16, 17]. As surgery continuously becomes more standardized and less invasive, TLDG is applied, with the ultimate goal of providing painless and scarless clinical treatment. However, natural orifice transluminal endoscopic surgery has clinical application limits because of many factors, so the introduction of single-port laparoscopy is considered to be an excessive stage of traditional laparoscopic surgery to natural orifice transluminal endoscopic surgery. Some scholars [18, 19] believe that the single-port laparoscopy is limited in the operation scope, lacks antitraction, and has narrow surgical field, which will lead to an enlarged umbilical incision, etc., so an additional operation hole is added by attempt for countertraction and reducing the difficulty of operation. The efficacy of the two-port laparoscopy method has been proved in previous literature [20, 21], because compared with traditional laparoscopy, patients who underwent the two-port laparoscopy could get off bed and had their indwelling tube removed sooner. As the laparoscopic surgical technique has become more mature, although with some defects such as long surgical time and learning curve, and fatiguing the operator during surgery, most physicians recognize that this surgical approach is as safe and thorough as the open radical gastrectomy. Foreign literature [22] has reported that the HALG was successfully performed to 2 cases for the first time by the abdominal wall suspension technology and the operator putting his left hand into the patient's abdominal cavity through the incision in the right lower abdomen, which was promoted and applied in the clinic as it fully combined the advantages of laparoscopy and open surgery.

The Clavien–Dindo classification system is currently the most definitive criterion internationally for evaluating the postoperative complications including gastric cancer [23]. This study provided the basis for preventing complications after radical gastrectomy by comparing the impact of two surgical methods on the severity of postoperative complications and analyzing the risk factors of postoperative complications. The results showed that age not less than 60 years old was an independent risk factor for postoperative complications, which might be related to the poor body tolerance of the elderly patients, a view that was supported by other scholars [24]; on the other hand, intraoperative blood loss not less than 180 ml was another risk factor, which was proved in the study by FESCO et al. [25]. The study also found that compared with DPLDG, the surgical time of HALG was obviously longer, but the length of surgical time was confirmed to be not significantly associated with the postoperative complications. However, the definition of postoperative adverse events after different surgeries by the Clavien–Dindo classification system might not be consistent and cause bias in the grading of complications in reality. This study was conducted with fewer cases and lack of research on patients' long-term efficacy, so large sample and multicenter prospective studies are still needed in the future to further validate the application value of the Clavien–Dindo classification system in the assessment of postoperative complications of gastric cancer, so as to make a risk assessment and early intervention treatment for the occurrence of postoperative complications in patients and maximize the surgical treatment effect.

In conclusion, patients tend to have grade II complications or complications with lower grade after surgery, and age not less than 60 years old and intraoperative blood loss not less than 180 ml are the independent risk factors that cause postoperative complications. Therefore, we should focus on the high-risk group for complications, take corresponding prevention and treatment measures, and reduce the incidence of postoperative complications.

## Figures and Tables

**Table 1:** Comparison of patients' baseline information between the two groups.

Item	DPLDG group (*n* = 386)	HALG group (*n* = 389)	*X* ^2^ (t)	*p*
Gender			0.101	0.750
Male	201 (52.07%)	207 (53.21%)		
Female	185 (47.93%)	182 (46.79%)		

Mean age ($\bar{x}\pm s$, **years old**)	58.47 ± 5.71	58.52 ± 5.28	0.127	0.899
BMI (kg/m^2)^	22.41 ± 1.62	22.43 ± 1.57	0.175	0.862
Tumor length (cm)	4.24 ± 1.03	4.29 ± 1.05	0.669	0.504

Pathological type				
Glandular carcinoma	214 (55.44%)	219 (56.30%)	0.058	0.810
Squamous cell carcinoma	114 (29.53%)	118 (30.33%)	0.059	0.808
Signet-ring cell carcinoma	58 (15.03%)	52 (13.37%)	0.438	0.508

Basic disease				
Diabetes	118 (30.57%)	120 (30.85%)	0.024	0.876
Hypertension	147 (38.08%)	153 (39.33%)	0.127	0.721
Kidney disease	121 (31.35%)	116 (29.82%)	0.213	0.645

Tumor location				
Antrum	207 (53.63%)	211 (54.24%)	0.030	0.864
Stomach body	146 (37.82%)	151 (38.82%)	0.081	0.776
Gastric angle	33 (8.55%)	27 (6.94%)	0.702	0.402

Range of gastrectomy			0.104	0.747
Total gastrectomy	195 (50.52%)	192 (49.36%)		
Partial gastrectomy	191 (49.48%)	197 (50.64%)		

TNM stage				
I	198 (51.30%)	201 (51.67%)	0.011	0.917
II	146 (37.82%)	149 (38.30%)	0.019	0.891
III	42 (10.88%)	39 (10.03%)	0.151	0.697

Differentiation type				
Low	189 (48.96%)	192 (49.36%)	0.012	0.913
Medium	152 (39.38%)	149 (38.30%)	0.094	0.759
High	45 (11.66%)	48 (12.34%)	0.085	0.770
Lymphatic metastasis rate (%)	46.11% (178/386)	46.53% (181/389)	0.014	0.908

Infiltrative depth				
T_1_	134 (34.72%)	129 (33.16%)	0.208	0.648
T_2_	95 (24.61%)	93 (23.91%)	0.052	0.819
T_3_	121 (31.35%)	126 (32.39%)	0.097	0.755
T_4a_	36 (9.33%)	41 (10.54%)	0.319	0.572
Number of lymph nodes submitted for inspection	24.41 ± 6.46	24.46 ± 6.48	0.108	0.914

**Table 2:** Comparison of patients' clinical effect indexes between the two groups $\left(\bar{x}\pm s\right)$.

Indicator	DPLDG group (*n* = 386)	HALG group (*n* = 389)	*t*	*p*
Surgical time (min)	173.25 ± 19.73	194.36 ± 23.65	13.497	≤0.001
Intraoperative blood loss (mL)	162.46 ± 25.36	171.35 ± 23.41	5.071	≤0.001
Incision length (cm)	5.73 ± 0.58	5.81 ± 0.62	1.854	0.064
Postoperative exhaust time (d)	2.67 ± 0.59	2.53 ± 0.62	3.220	0.001
Time to return to full-liquid diet for the first time after surgery (d)	2.36 ± 0.36	2.41 ± 0.43	1.754	0.080
Hospital stay after surgery (d)	9.35 ± .86	9.42 ± 1.73	0.543	0.588
Off-bed ambulation time after surgery (d)	2.74 ± 0.61	2.68 ± 0.58	1.403	0.161
Number of lymph nodes being dissected (*n*)	28.43 ± 3.27	28.51 ± 3.16	0.346	0.729
Time of removing the abdominal drainage tube (h)	4.15 ± 1.42	4.23 ± 1.38	0.795	0.427

NRS score (points)				
6 h after surgery	4.72 ± 0.82	4.68 ± 1.03	0.598	0.550
12 h after surgery	3.54 ± 0.75	3.62 ± 0.68	1.556	0.120
24 h after surgery	2.21 ± 0.53	2.28 ± 0.58	1.753	0.080
2 d after surgery	1.47 ± 0.45	1.51 ± 0.35	1.382	0.167

Aesthetic scoring (cases)				
Low	236 (61.14%)	241 (61.95%)	0.054	0.816
Medium	85 (22.02%)	87 (22.37%)	0.013	0.908
High	65 (16.84%)	61 (15.68%)	0.191	0.662

**Table 3:** Comparison of patients' Clavien–Dindo grades of postoperative complications between the two groups [*n* (%)].

Complication type	DPLDG group (*n* = 386)	HALG group (*n* = 389)	*X* ^2^	*p*
≤II	27 (36.00%)	53 (51.46%)	4.190	0.041
IIIa	15 (20.00%)	18 (17.48%)	0.183	0.669
IIIb	16 (21.33%)	12 (11.65%)	3.524	0.060
IVa	11 (14.67%)	10 (9.71%)	1.025	0.311
IVb	4 (5.33%)	7 (6.80%)	0.160	0.689
V	2 (2.67%)	3 (2.91%)	0.010	0.922
Overall complication incidence	75 (19.43%)	103 (26.48%)	5.440	0.020

**Table 4:** Single-factor and multivariate analysis of risk factors of postoperative complications in patients of both groups.

Item	Single-factor analysis		Multivariate analysis	
	Overall incidence rate of complications (%)	*p*	OR value (95% CI)	*p*
Gender		0.426		
Male	12.10			
Female	10.84			

Age		0.007	1	0.037
<60 years old	9.29		1.346 (1.048, 1.761)	
≥60 years old	13.68			

Range of gastrectomy		0.339		
Total gastrectomy	12.26			
Partial gastrectomy	10.71			

Surgical method		0.022	1	0.028
DPLDG	9.42		0.742 (0.562, 0.982)	
HALG	13.16			

Tumor length		0.010	1	0.462
<5 cm	9.55		1.137 (0.852, 1.525)	
≥5 cm	13.42			

Pathological stage		0.003	1	0.871
Stage I	9.06		1.051 (0.646, 1.751)	
Stages II-III	13.83			

Intraoperative blood loss (ml)		<0.001	1	<0.001
<180 ml	16.5		1.635 (1.249, 2.14)	
≥180 ml	27.4			

Number of lymph nodes submitted for inspection (*n*)		0.633		
<25	11.10			
≥25	11.87			

Surgical time		0.736		
Less than 180 min	12.31	<0.001		
No less than 180 min	11.27			

## Data Availability

The datasets used and/or analyzed during the present study are available from the corresponding author upon reasonable request.
